# Anti-Methicillin-Resistant *Staphylococcus aureus* Efficacy of Layer-by-Layer Silver Nanoparticle/Polyacrylic Acid-Coated Titanium Using an In-House Dip Coater

**DOI:** 10.3390/polym17030333

**Published:** 2025-01-25

**Authors:** Julinthip Puttawong, Mingkwan Yingkajorn, Pasarat Khongkow, Soracha D. Thamphiwatana, Tonghathai Phairatana

**Affiliations:** 1 Department of Biomedical Sciences and Biomedical Engineering, Faculty of Medicine, Prince of Songkla University, Hat Yai, Songkhla 90110, Thailand; julinthip.p@gmail.com (J.P.); pasarat.k@psu.ac.th (P.K.); 2 Department of Pathology, Faculty of Medicine, Prince of Songkla University, Hat Yai, Songkhla 90110, Thailand; mingkwan.y@psu.ac.th; 3 Institute of Biomedical Engineering, Faculty of Medicine, Prince of Songkla University, Hat Yai, Songkhla 90110, Thailand; 4 Department of Biomedical Engineering, Faculty of Engineering, Mahidol University, Nakornpathom 73170, Thailand; 5 International School of Engineering (ISE), Faculty of Engineering, Chulalongkorn University, Bangkok 10330, Thailand

**Keywords:** arthroplasty-associated infection, layer-by-layer, MRSA, polyacrylic acid, silver nanoparticles

## Abstract

The emergence of methicillin-resistant *Staphylococcus aureus* (MRSA) is still posing a global challenge in healthcare settings. This bacterial strain is a cause of severe periprosthetic infection, thereby impairing the success of implant insertion. To address this issue, implant surface modification is required. Herein, we developed a novel multilayered silver nanoparticle/polyacrylic acid-coated Ti plate (AgNPs/PAA/Ti) using an in-house dip coater. AgNPs were synthesized and characterized. The dip-coating process was optimized based on the dipping rate, evaporation time, and coating cycle number. Uniform and reproducible coatings were achieved on Ti surfaces, with consistency verified through SEM analysis. The average size of the AgNPs was approximately 36.50 ± 0.80 nm with a PDI of 0.443 ± 0.025, and the zeta potential was measured at around –23.3 ± 2.0 mV. The maximum coating thickness of 83.5 ± 1.3 µm was observed at 15 cycles of dip coating. Moreover, our developed AgNPs/PAA/Ti plate showed both antimicrobial and biofilm-resistant performance, while also exhibiting enhanced biocompatibility with cultured MG63 osteosarcoma cells, maintaining cell viability greater than 70%. We envisage that this material holds significant promise as a candidate for medical implant devices, offering protection against MRSA-associated infection at insertion sites with low vascularity in the future.

## 1. Introduction

Methicillin-resistant *Staphylococcus aureus* (MRSA) has emerged as a serious causative agent of periprosthetic infection worldwide. The ability of this bacterial strain to form antibiotic-resistant biofilms poses a significant challenge for treatment, thereby increasing the mortality risk among hospitalized patients. Numerous studies have reported the detrimental effects of MRSA colonization on patients. Previous research has shown that MRSA can grow on various devices, including central venous catheters, polystyrene-containing devices [1], knee implants [2], breast implants [3], cardiovascular electronic devices [4], and heart valves [5]. After implantation, cleaning these inserted devices becomes increasingly difficult. Moreover, MRSA colonization and its biofilm are typically resistant to therapeutic antibiotics. The detachment of bacterial cells or biofilm fragments to other parts of the body can lead to severe systemic infections. Periprosthetic infections significantly impact patients by incurring high costs for implant replacement, prolonging wound healing, and increasing the risk of re-infection [6,7]. To address these challenges, there is a critical need for innovative antibacterial and antibiofilm materials that can be incorporated into medical implant devices to prevent such complications. Biofilms create a protective barrier that shields bacteria from antibiotics and the host immune response, making infections difficult to eradicate. Current treatment approaches, including prolonged antibiotic regimens, often lead to bacteria-resistant and recurrent infections [8]. These limitations underscore the pressing need for innovative solutions. A transition to antibacterial materials for orthopedic implants could address these challenges more effectively. Such materials, designed to inhibit bacterial colonization and biofilm formation directly at the implant site, offer a promising alternative to traditional treatments.

Over recent decades, nanotechnology has advanced the development of materials to prevent bacterial colonization on medical devices. Nanoparticles possess a large surface area and high dispersive properties, which enhance their ability to effectively reach targets including metal nanoparticles [9]. Nanoparticles exhibit various modes of action, including bacterial membrane disruption, reactive oxygen species (ROS) formation, inhibition of DNA/RNA replication, and DNA destruction [10,11]. Among them, silver nanoparticles (AgNPs) are particularly well known for their antimicrobial activity against both Gram-positive and Gram-negative bacteria. Studies have demonstrated their effectiveness against multidrug-resistant bacteria such as MRSA, overcoming bacterial resistance [12,13]. AgNPs act by either binding to and disrupting the bacterial membrane or entering the cell to induce damage through ROS generation and DNA and ribosome destruction [14,15]. However, in vivo studies have shown that lower concentrations of AgNPs can exert beneficial effects on mammalian cells, while higher concentrations tend to cause damage [16,17]. Their antimicrobial functionality can also be enhanced through surface modification. AgNP-coated materials are particularly effective in reducing bacterial adhesion and biofilm formation, making them ideal for use in implant coating. These coatings disrupt bacterial membranes via ion release and direct interactions while maintaining biocompatibility, offering significant potential for preventing infection in biomedical applications, especially diverse antibacterial implant technologies [18,19,20].

Orthopedic implants play a crucial role in modern medical treatments, providing stability and support and promoting healing in various bone and joint-related conditions. However, their long-term success is often hindered by complications such as foreign body reactions, granuloma formation, and allergic responses caused by the degradation products of conventional materials [21]. To overcome these challenges, researchers have explored bioabsorbable and bioactive materials, such as polylactic acid, polyglycolic acid, polyacrylic acid, and their copolymers [22]. Among these, polyacrylic acid (PAA) has emerged as a promising candidate for coating orthopedic implants due to its bioactivity, biocompatibility, and biodegradability. This material has the potential to address complex issues associated with osteochondral repair and improve the fixation and repair processes of tendons and ligaments. PAA coatings create a bioactive interface between the implant and surrounding tissues, promoting better integration, reducing foreign body interactions, and supporting the healing and regeneration of critical musculoskeletal structures. The ability to tailor the biodegradation rate and modify the surface properties further enhances the versatility of PAA coatings for orthopedic applications. As biomedical engineering advances, the incorporation of PAA-based materials into orthopedic implants offers innovative solutions to complex clinical challenges [23]. These materials not only expand the options available to surgeons but also hold great promise for improving patient outcomes and enhancing the long-term success of these vital medical devices [22,24].

Traditionally, the use of antibiotics and antimicrobial agents has contributed to the rise in antibiotic-resistant bacterial strains and various adverse side effects [25]. Coatings with single antibacterial mechanisms lack multi-functionality, providing limited effectiveness against resistant strains such as MRSA. There is a need for coatings that integrate multiple antibacterial strategies, such as combining contact-killing and release-killing properties with multi-mechanistic action to simultaneously target biofilm formation, adhesion, and bacterial viability [26]. To address these challenges, researchers have shifted their focus toward developing novel surface coatings for implantable materials to effectively prevent bacterial attachment and biofilm formation. One promising approach is the creation of a multifunctional surface coating that combines non-adhesive and antimicrobial properties. These coatings often incorporate antimicrobial peptides, which target bacteria while minimizing the risk of resistance development [27]. Another innovative strategy involves engineering unique micro- and nano-topographies on the implant surface. These topographical modifications alter the material’s physical and chemical properties, making it less conducive to bacterial colonization and biofilm formation. These advanced surface modification techniques offer several key advantages, including localized antimicrobial action, reduced systemic side effects, and the potential for regeneration or reapplication. However, some sophisticated techniques, such as covalent binding processes or photochemical reactions, can be technically complex and challenging to scale up. In contrast, simpler methods such as dip coating or spray coating provide practical and scalable solutions for surface modification. Additionally, layer-by-layer (LBL) assembly is gaining attention as a versatile technique for creating multilayer coatings with synergistic effects. This approach enables precise control over the composition and thickness of coatings, enhancing their effectiveness in combating bacterial infections and promoting the integration of implants [27,28,29].

Despite advancements in surface modification techniques, some coatings still face limitations, such as reduced long-term stability or insufficient antimicrobial efficacy. Therefore, further research focused on optimizing the protocols for surface modification is essential to enhance the performance, scalability, and practical application of these coatings in clinical settings. Such a combination of multilayers could synergistically enhance bactericidal effects against antibiotic-resistant bacteria. This integrated strategy not only enhances the antibacterial effect but also provides a more robust and adaptable solution for combating implant-associated infections, paving the way for safer and more reliable implant technologies.

**Figure 1:**
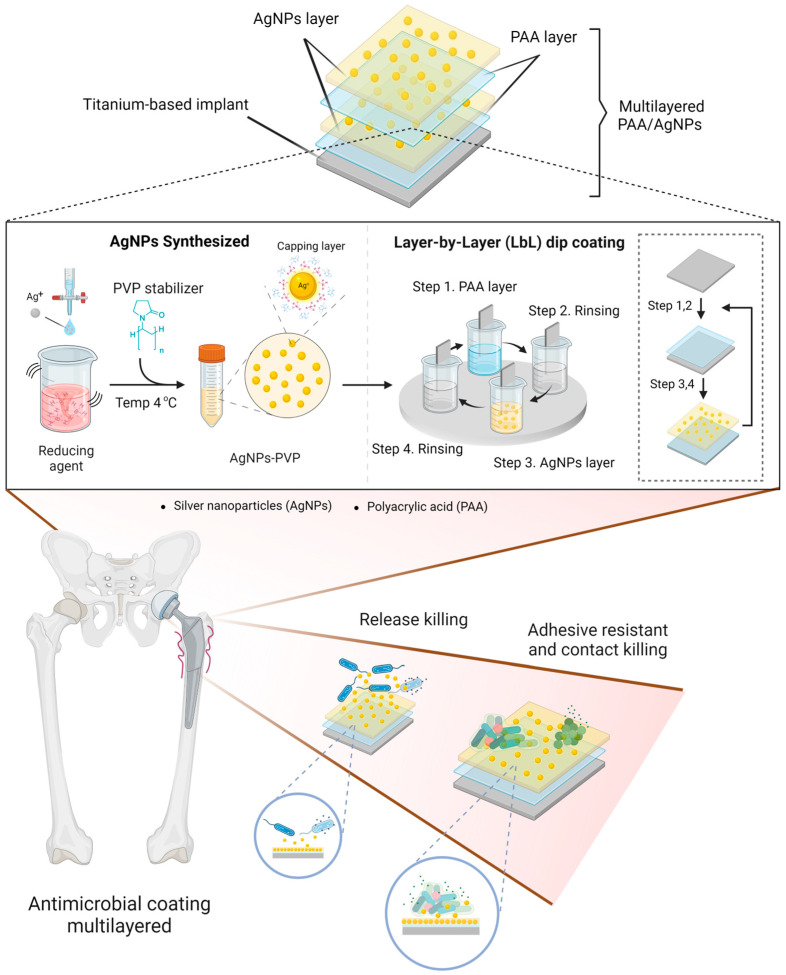
Schematic of the research design for synthesized AgNP/PAA-coated orthopedic implants using the layer-by-layer dip-coating method (created by BioRender.com accessed on 30 November 2024).

In this study, we developed a novel protocol for silver nanoparticle (AgNP)-embedded multilayered polyacrylic acid (PAA) to treat MRSA-associated periprosthetic infections and prevent biofilm formation. As shown in Figure 1, AgNPs were synthesized using polyvinylpyrrolidone (PVP) as a stabilizer and subsequently characterized. The modification of the AgNP/PAA-Ti surface was performed in a controlled manner using a layer-by-layer (LbL) dip-coating method with an in-house dip coater. We thoroughly characterized and evaluated the physicochemical properties of the developed coating surface. Finally, we demonstrated that the AgNP/PAA-coated orthopedic implant effectively inhibited MRSA growth, exhibited antibiofilm activity, and maintained biocompatibility.

## 2. Materials and Methods

### 2.1. Reagents

Polyacrylic acid (PAA, MW ≈ 50,000 Da, aqueous solution), sodium borohydride (NaBH_4_), silver nitrate (AgNO_3_), and polyvinylpyrrolidone (PVP) were purchased from Sigma-Aldrich (St. Louis, MO, USA). The LIVE/DEAD™ cell imaging kit (488/570) was supplied by Thermo-Fisher Scientific (Waltham, MA, USA). Dulbecco’s Modified Eagle Medium (DMEM) and phosphate-buffer solution (PBS) were bought from Thermo Fisher Scientific (Waltham, MA, USA). The MTT assay kit was obtained from Abcam (Cambridge, MA, USA). MG-63 osteosarcoma cells (EGFR+, epidermal growth factor receptor-positive) and methicillin-resistant *Staphylococcus aureus* were provided by the Department of Biomedical Sciences and Biomedical Engineering, Faculty of Medicine, Prince of Songkla University. Bacterial media, e.g., nutrient agar and nutrient broth, were supplied by HiMedia Laboratories Private Limited (Thane, Maharashtra, India). All aqueous solutions were prepared using Milli-Q purified water (resistivity ≥ 18 MΩ cm, at 25 °C; Millipore, Burlington, MA, USA).

### 2.2. Synthesis of Silver Nanoparticles

Silver nanoparticles (AgNPs) were chemically synthesized through a chemical reduction process. Firstly, a 2 mM sodium borohydride (NaBH_4_) solution was freshly prepared by dissolving in cooled distilled water to a final volume of 30 mL. The NaBH_4_ solution was then stirred at room temperature and 1400 rpm until it reached 40 °C. Secondly, 10 mL of 1 mM silver nitrate was added to the NaBH_4_ solution dropwise at a rate of 1 drop/second. The two solutions were allowed to react at room temperature for 15 min. Finally, 4 mL of 0.3% (*w*/*v*) PVP stabilizer was added to the yellow silver nanoparticle solution. This reaction step was performed in the dark at 4 °C. The synthesized AgNPs were characterized using several techniques. Dynamic light scattering (DLS) and zeta potential analysis were employed to evaluate the size distribution and stability of nanoparticles in the polymeric solution, respectively. A zetasizer instrument (Nano ZS, Malvern, Worcestershire, UK) was used to measure the intensity and zeta potential in a disposable cuvette at a scattering angle of 173° (NIBS default), with a temperature of 25 °C and an equilibration time of 30 s. The microscopic morphology of the AgNPs was examined using a transmission electron microscope (TEM, JEOL JEM-2010, Tokyo, Japan). The absorbance of the AgNPs was measured using UV–visible spectroscopy using microplate readers (Infinite 200Pro, Tecan Group Ltd., Mannedorf, Switzerland).

### 2.3. Layer-by-Layer Dip Coating

Ti plates were initially cleaned and polished with Wenol™ and isopropyl alcohol (IPA), and then sonicated in acetone for 1 min, rinsed with DI water, and dried with nitrogen gas. Before coating, the plates were pretreated with a plasma cleaner. Layer-by-layer (LbL) dip coating was performed using an in-house dip coater. In one cycle, the plate was coated using a sol–gel solution containing 1.44 mg/mL of polyacrylic acid (PAA) dissolved in DI water and followed by a AgNP solution. After each cycle, the plates were air-dried at room temperature before proceeding to the next cycle. To optimize performance, key dip-coating parameters were investigated, focusing on achieving maximum thickness: dip-coating rate (2, 3, 5 rpm), evaporation time (2, 5, 10 min), and number of cycles (5, 15, 25 cycles). A digital thickness monitor (Nikon Digimicro Stand MS-21, Tokyo, Japan) with a Digimicro MFC-101A was used to examine the thickness of the coated Ti samples.

### 2.4. Characterization of AgNP/PAA/Ti Plate Coating

The surface of the dip-coated Ti plates modified with AgNPs/PAA was characterized. A field emission scanning electron microscope (Apreo FE-SEM, FEI Company, Eindhoven, The Netherlands) was used to examine surface morphology and nanoparticle shapes. The SEM was operated at 5–10 keV to generate images of metal and polymeric nanoparticles embedded in the polymer matrix through valence electron scattering. Additionally, an energy-dispersive X-ray spectrometer (EDS, Hitachi TM3030, Tokyo, Japan) was employed to analyze the chemical composition of the samples. Atomic force microscopy (AFM, Nanosurf Flux model, Liestal, Switzerland) was also used to assess the surface roughness characteristics of metal and polymeric nanoparticles, with the AFM probe scanning the surface to produce high-resolution 2D and 3D topographical images at the nanometer scale. Furthermore, the hydrophobicity and hydrophilicity of the modified Ti plates were assessed using a static optical contact angle analyzer (OCA25, Data Physics, Filderstadt, Germany) with sessile and pendant drop methods to measure surface tension.

### 2.5. Bacterial Culture

Methicillin-resistant *Staphylococcus aureus* ATCC BAA1720 was used in this experiment. First, bacteria strain stored in 40% glycerol at −80 °C was streaked onto sheep blood agar and incubated at 37 °C for 1 day. Following this, a monoclonal strain was transferred into 1 mL of nutrient broth (Mueller-Hinton broth, MHB) liquid medium in a microtube and incubated in a shaker at 37 °C and 200 rpm for 6 h. Finally, the turbidity of the bacteria cultures was then adjusted to 0.5 McFarland standard (1.5 × 10^8^ colony-forming unit (CFU)/mL) using 0.85% normal saline (NSS) and subsequently diluted to 1 × 10^6^ CFU/mL.

### 2.6. In Vitro Antimicrobial Assay

Vancomycin was selected as the control antibiotic in this study as it is widely recognized as the gold standard for antimicrobial susceptibility testing, particularly for MRSA [30,31]. Antimicrobial assays were performed using the microdilution method. In vitro antimicrobial properties, minimum inhibitory concentration (MIC), and minimum bactericidal concentration (MBC) were determined. To determine the MIC, the AgNP solution (50 µL) was diluted to 10 concentrations via a serial dilution in a 96-well plate using MHB. Subsequently, 50 µL of MRSA and 100 µL of MHB nutrient broth were added to each well, establishing sterile controls (vancomycin without bacteria) and growth controls (bacteria without vancomycin). The plate was incubated overnight at 37 °C. The growth of bacteria was assessed by measuring optical density (OD) at a 600 nm wavelength using a microplate reader. The MBC was determined using the plating technique, where each solution from the wells was directly plated onto nutrient agar to determine the number of viable bacteria.

### 2.7. Antibiofilm Investigation

The AgNP/PAA-coated titanium plate was incubated with 500 µL of methicillin-resistant *S. aureus* culture (10^6^ CFU/mL) in Luria broth (LB) medium with 1% glucose and incubated overnight at 37 °C to allow biofilm formation on the surface. The biofilm-covered Ti plate was then washed three times with PBS at pH 7.0 to remove non-adhesive planktonic bacteria and dried. The bacterial biofilm was fixed with 2.5% glutaraldehyde and dried at room temperature for 20 min. For SEM analysis, a graded ethanol series (25%, 50%, 75%, and 100%) was used to dehydrate the biofilm, replacing water and minimizing artifacts caused by rapid dehydration. The biofilm morphology was then examined using a scanning electron microscope (SEM, Hitachi TM3030, Tokyo, Japan).

### 2.8. Cytotoxicity Testing

To examine the cytotoxic effects of AgNPs, both quantitative and qualitative methods were used for a comprehensive evaluation. The quantitative analysis was carried out using the MTT assay to assess cell viability through absorbance measurement, while the qualitative analysis was performed using LIVE/DEAD staining to visually confirm cytotoxic effects. The MG-63 cells were cultured in Dulbecco’s Modified Eagle Medium (DMEM) supplemented with 10% Fetal Bovine Serum (FBS), 1% Penicillin–Streptomycin (Penstrep), and 1% L-glutamine. The cell cultures were incubated under a humidified 5% CO_2_ atmosphere overnight at 37 °C. The cells were trypsinized with 0.05% trypsin and 0.02% ethylenediaminetetraacetic acid (EDTA) and subcultured into new plates every 1–2 days when the confluency reached 80%. The medium was replaced every 1–2 days. Once the cell proliferation of MG63 cells reached 80% confluence again, the cells were trypsinized, the reaction was stopped with DMEM, and the medium was diluted to 1 × 10^5^ cells/mL.

For the MTT assay, AgNPs in PVP-stabilized form were used for testing. The MG63 cells were planted at a density of 5000 cells per well in a 96-well cell culture plate. After 24 h, the cells were exposed to varying doses of AgNPs. Following this, 10 µL of 3-(4,5-dimethylthiazol-2-yl)-2,5-diphenyltetrazolium bromide (yellow tetrazolium salt, MTT) in PBS (5 mg/mL) stock was added to each well. After that, the plates were incubated in a CO_2_ incubator at 37 °C for 4 h in the dark. After incubation, the metabolically active cells reduced MTT to insoluble formazan crystals. The MTT-containing medium was removed, the formazan crystals were dissolved in 110 µL of DMSO for 15 to 20 min at 37 °C, and a purple color developed. Cell viability was assessed by measuring the absorbance of the dissolved formazan using a microtiter plate reader at 570 nm wavelength. AgNPs were not used in the control experiment. The measured absorbance was directly correlated with cell viability and expressed as a percentage, with 100% representing the viability of control cells.

Cytotoxicity effects were qualitatively determined using a LIVE/DEATH^TM^ cell imaging kit (Invitrogen) at 24 h and 72 h time points. AgNP-coated Ti plates (W × L × H = 1.0 × 1.5 × 0.05) were used as testing materials and the polished Ti plate without coating was used as a control. The number of living cells was proportional to the number of cells with calcein green fluorescence after conversion via intracellular esterases. First, the medium was removed from the cells. Second, staining solution (100–200 µL) was directly added into the cells at a 250:250 DMEM/dye volume ratio. The stain was incubated at 20–25 °C for 30 min before examination under a Lionheart FX fluorescence microscope (Lionheart FX, BioTek Instrument, Santa Clara, CA, USA) for cell imaging. The excitation wavelengths of green fluorescent protein (GFP) and Texas red (sulforhodamine 101 acid chloride) were 469/525 nm and 58/647 nm, respectively.

### 2.9. Statistical Analysis

All experiments determined the significance of the variations in mean values. Standard deviation (SD) was indicated using error bars. GraphPad Prism™ (Version 10.0) software was used for the statistical analyses (mean ± SD, n = 3). One-way ANOVA was utilized to compare the means of two or more independent groups, followed by Tukey’s post hoc test for multiple comparisons when significant differences were observed. Statistical significance was determined at *p* < 0.05. Normality was assessed to ensure the assumptions of the test were met.

## 3. Results and Discussion

### 3.1. Characterization of Silver Nanoparticles

**Figure 2:**
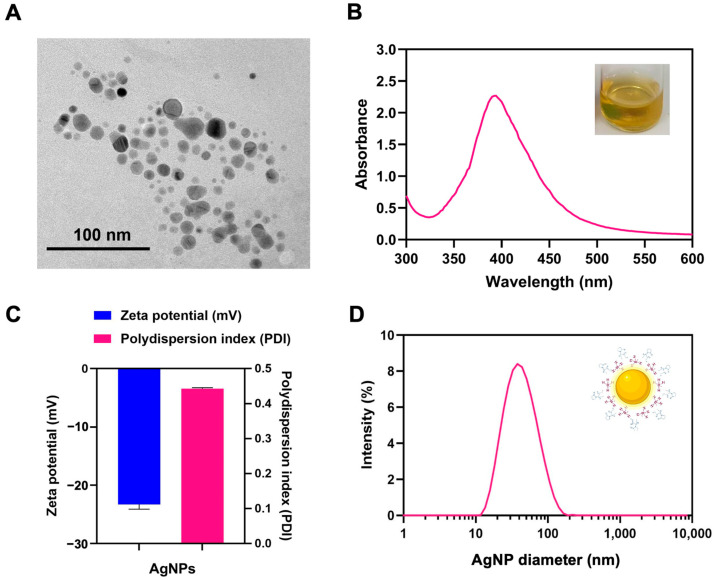
AgNP characterization. (**A**) TEM image. (**B**) UV–visible spectroscopy analysis of AgNPs showing a prominent surface plasmon resonance peak around 400 nm. The inset displays the appearance of the AgNP solution with a yellowish color. (**C**) The zeta potential (left) and PDI (right) of AgNPs. (**D**) Dynamic light scattering (DLS) analysis showing a narrow distribution, indicating the uniform size of AgNPs.

The TEM image of the synthesized AgNPs (Figure 2A) revealed a spherical shape with a size ranging from 15 to 40 nm. UV-Vis absorption spectra (Figure 2B) are a key tool for characterizing AgNPs as they reveal their optical properties, including their characteristic surface plasmon resonance (SPR). The results confirmed that the absorbance peak of AgNPs occurred at around 400 nm, indicating that surface plasmon excitation occurred [32].

The size and zeta potential of the synthesized AgNPs were measured using dynamic light scattering (DLS) analysis. These results revealed that the zeta potential of the synthesized AgNPs was approximately −23.3 ± 2.0 mV, with a polydispersity index (PDI) of 0.443 ± 0.025 (Figure 2C), and the average size of the AgNPs was approximately 36.50 ± 0.8 nm (Figure 2D). These results indicate that the AgNPs provided uniformity and stable dispersion in aqueous or polar media. The zeta potential reflects the surface charge of the nanoparticles, demonstrating their electrostatic stability in colloidal systems, while the PDI confirms the successful synthesis of uniformly dispersed, monodisperse AgNPs. This is because polyvinylpyrrolidone (PVP) provided a unique chemical structure when used as a stabilizing agent for AgNP synthesis; it features a hydrophilic backbone and functional amide groups, enables the prevention of agglomeration by forming a steric barrier around nanoparticles through adsorption on their surface, contributes to nanoparticle size control by regulating nucleation and growth during synthesis, and interacts strongly with Ag^+^ ions to ensure stable dispersion in aqueous or polar media.

### 3.2. Coating Characterization

#### 3.2.1. LBL-Based Coating Optimization

**Figure 3:**
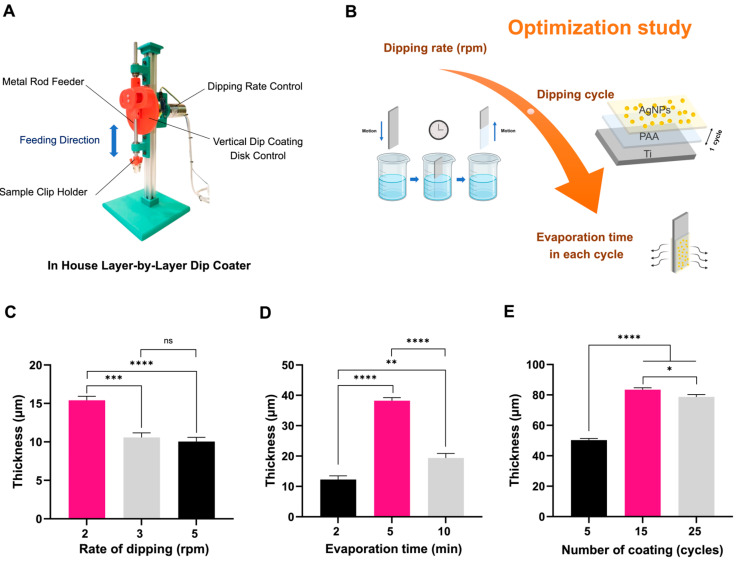
Characterization of LBL-based AgNP/PAA/Ti coating process. (**A**) Photo of the in-house dip coater showing its components. (**B**) Schematic of parameter optimization. The results for each parameter based on thickness: (**C**) dipping rate, (**D**) evaporation time, and (**E**) number of coating cycles. Each value represents the mean ± standard deviation (SD) with triplicates (n = 3). * indicates a difference at *p* < 0.05, ** at *p* < 0.01, *** at *p* < 0.001, and **** at *p* < 0.0001, and “ns” is no significance.

In this work, an in-house dip coater was developed that could control the dip-coating rotation rate and the number of coating cycles using an Arduino controller to ensure precise control over coating thickness and uniformity. The photo of our in-house coater presented in Figure 3A shows the components of the machine, which was designed with three clips to attach the Ti plates. The process of Ti plate modification is illustrated in Figure 3B. This involved submerging the Ti plate into a coating solution containing PAA and AgNPs. The PAA serves as a binding agent for the AgNP layer. Its carboxylic groups enhance adhesion to the titanium substrate while allowing ionic interactions with the AgNPs [33,34]. Additionally, PAA layers contribute to hydrophilicity, promoting compatibility with biological tissues. Then, the AgNP/PAA-coated Ti in each cycle was evaporated at room temperature. To achieve the effectiveness of the coating, the involved fabrication parameters were optimized, including the rate of dip-coating, the evaporation times, and the number of coating cycles. The results are shown in Figure 3C, Figure 3D, and Figure 3E, respectively. As shown in Figure 3C, the thickness was inversely related to the rate of dipping. At slower dipping rates of 2 rounds per minute (rpm), the Ti plate remained in the solution for a longer duration, leading to a higher accumulation of AgNPs. Consequently, a thicker coating was obtained. In contrast, faster dipping rates of 3 and 5 rpm resulted in thinner coatings due to reduced interaction time with the solution. Using the optimized dipping rate for the next step to examine evaporation optimization, the results (Figure 3D) showed that the thickness peaked at 5 min, indicating effective solvent evaporation and layer consolidation. This could be explained by the shorter time at 2 min leading to incomplete drying, while the longer time at 10 min may cause over-consolidation, reducing the efficiency of layer deposition. Thus, the 5 min evaporation condition was chosen as it struck a balance between rapid processing and optimal thickness, ensuring robust coatings with strong antimicrobial potential [35,36]. Multiple cycles ensured a higher payload of AgNPs, allowing for prolonged Ag^+^ release [37]. As shown in Figure 3E, the thickness of the film increased linearly with the number of coating cycles. The highest thickness observed in this study was 83.5 ± 1.3 µm at 15 cycles, while a moderate thickness of 48.6 ± 0.5 µm was achieved at 5 cycles. Saturation effects occurred beyond 25 cycles, limiting further deposition efficiency. Therefore, 15 cycles were chosen as the optimal balance between adequate thickness and manageable processing time.

#### 3.2.2. Surface Characterization

**Figure 4:**
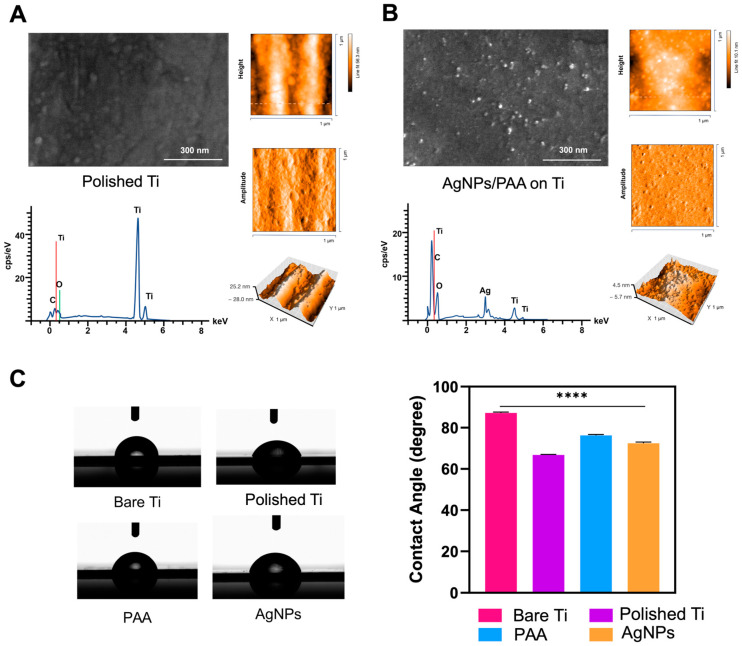
Surface characterization. SEM (200 k magnification) and AFM images of (**A**) polished Ti plate and (**B**) AgNP/PAA-coated Ti plate. (**C**) Contact angles of Ti plates with different surface modifications. Each value is presented as the mean ± SD (n = 3). **** indicates a difference at *p* < 0.0001.

After optimizing the key factors for AgNP/PAA modification, the surface morphology of the optimally coated Ti plate was analyzed and compared to the uncoated Ti plate using 2D and 3D imaging techniques, specifically SEM and AFM, respectively. The SEM image in Figure 4A shows the uncoated surface (polished Ti as a control) with typical grinding marks, while Figure 4B depicts the surface morphology of AgNP/PAA-coated Ti implants, where the AgNPs are clearly visible as white dots. EDS spectra were analyzed to confirm the successful deposition of AgNPs on the Ti surface. Distinct peaks corresponding to silver validated the presence of the coating, while titanium peaks confirmed the underlying substrate. The intensity of the silver peaks correlated with the nanoparticle density on the surface [38,39]. Additionally, peaks of oxygen and carbon were identified, likely originating from the polyacrylic acid (PAA) binder and residual oxides. The presence of oxygen suggested that the LbL process produces a hybrid organic–inorganic coating, enhancing both biocompatibility and adhesion [40]. Clear differences in surface texture were observed, with the coated surface appearing distinctly altered due to the coating process. AFM analysis revealed 3D nanoscale surface features, highlighting a unique surface pattern and nanoparticle distribution. The accompanying color scale bar indicated the height profile, with Figure 4B showing that the coated surface exhibited a significantly rougher texture. The root mean square roughness (Sq) values were 1.64 nm for the AgNP/PAA-coated Ti and 11.57 nm for the polished Ti. The lower Sq value of the AgNP/PAA-coated surface indicated that it was smoother than the polished Ti, demonstrating the success of the coating. Furthermore, this reduced roughness was attributed to the deposition of AgNP clusters embedded within the polymer matrix, which created a hierarchical surface structure.

The contact angle has been widely used to evaluate the efficacy of surface modification techniques [41]. The contact angles (in degrees) for different surface modifications of titanium are shown in Figure 4C. The surface hydrophilicity and hydrophobicity varied across the four types of Ti surface, i.e., bare Ti, polished Ti, Ti coated with PAA, and Ti coated with AgNPs. Figure 4C (left) presents representative water droplet images, demonstrating the contact angles for each surface type. These images correspond to the graph in Figure 4C (right) and illustrate differences in wettability. The measured contact angles were 87.2° ± 0.4° for bare Ti, 66.8° ± 0.2° for polished Ti, 76.3° ± 0.4° for PAA-coated Ti, and 72.5° ± 0.6° for AgNP-coated Ti. The bare Ti surface exhibited the highest contact angle, indicating a hydrophobic surface. In contrast, polished Ti and coated surfaces demonstrated reduced contact angles, reflecting improved wettability due to surface modifications. These results underscore the critical role of coating and surface treatments in enhancing the wettability characteristics of Ti. Although Ti is inherently biocompatible, its hydrophobic nature is less favorable for promoting cellular adhesion and antibacterial properties [42]. Our findings emphasize the importance of surface modifications to enhance both hydrophilicity and biological functionality. Notably, highly hydrophilic surfaces, while potentially discouraging bacterial adhesion, may also impede the release of Ag^+^ ions. The observed contact angles suggest a balance, ensuring sufficient hydrophilicity to support cellular interactions while maintaining effective Ag^+^ ion release for antimicrobial action [43].

### 3.3. Antibacterial Performances

#### 3.3.1. MIC and MBC Determination

**Figure 5:**
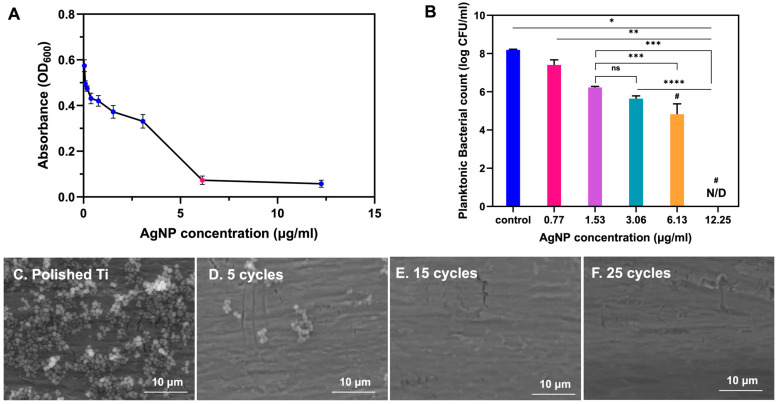
Efficacy of AgNP concentrations against MRSA activities. (**A**) Minimum inhibitory concentration (MIC) measured with absorbance at OD_600_. (**B**) Minimum bactericidal concentration (MBC) represented as bacterial counts (log CFU/mL) after treatment with varying AgNP concentrations, where no bacterial colonies were detected (denoted as ‘N/D’). Data are presented as mean ± SD (n = 3). * indicates a difference at *p* < 0.05, ** at *p* < 0.001, *** at *p* < 0.005, and ****, # at *p* < 0.0001, and “ns” is no significance. SEM images (4000×) showing inhibition of MRSA biofilm formation at different coating cycles: (**C**) polished Ti, (**D**) 5 cycles, (**E**) 15 cycles, and (**F**) 25 cycles.

AgNPs have emerged as a potent antimicrobial agent due to their broad-spectrum activity against a variety of pathogens, including multidrug-resistant organisms such as MRSA [11,12]. Our study evaluated the antimicrobial activity of AgNPs against MRSA by determining the minimum inhibitory concentration (MIC) through absorbance measurements at a wavelength of 600 nm (OD_600_). As shown in Figure 5A, the MIC was identified at 3.06 µg/mL AgNPs, where a significant decrease in absorbance was observed, indicating the effective inhibition of MRSA growth. Figure 5B illustrates the minimum bactericidal concentration (MBC), identified through a bar graph showing bacterial counts (log CFU/mL) after treatment with varying AgNP concentrations. A significant reduction in bacterial counts highlights the bactericidal effect of AgNPs. The MBC, defined as the concentration at which no bacterial colonies were detected (denoted as ‘N/D’), was determined for AgNPs against MRSA. The minimum concentration required to eliminate 99.9% of the bacterial population was found to be 12.25 µg/mL. The AgNPs adhered to bacterial membranes, causing structural damage and increased permeability. This leads to the leakage of cellular contents and eventual cell death [44,45]. These findings highlighted the dual efficacy of AgNPs in both inhibiting bacterial growth and achieving bactericidal effects at relatively low concentrations, making them promising for orthopedic applications.

#### 3.3.2. Antibiofilm Performance

Currently, biofilm formation can be prevented through two primary approaches, namely enhancing biomaterial coatings with antimicrobial agents and modifying implant surfaces to exhibit antiadhesive properties [46]. AgNPs have demonstrated significant potential in biofilm prevention by effectively inhibiting initial microbial adhesion and disrupting biofilm initiation processes. However, the design of AgNP coatings for implants remains a significant challenge. In this study, four different coating cycles were investigated to assess the antibiofilm performance of AgNP/PAA-coated Ti plates: polished Ti, 5 cycles, 15 cycles, and 25 cycles. The corresponding SEM images are shown in Figure 5C to Figure 5E, respectively. The SEM results revealed that the biofilm exhibited dense clusters of bacteria distributed across the untreated surface, as shown in Figure 5C. In contrast, the surface coated with five cycles and exposed to MRSA showed a significant reduction in biofilm formation compared to the untreated surface. Bacterial clusters were sparse, indicating the coating’s inhibitory effects, as shown in Figure 5D. The surfaces coated with 15 cycles in Figure 5E and 25 cycles in Figure 5F showed enhanced antibacterial coatings after treatment. These surfaces exhibited a complete absence of visible bacterial biofilm, confirming the coating’s effectiveness in preventing MRSA colonization. Therefore, the AgNP/PAA/Ti plate with 15 coating cycles proved to be the most effective against MRSA biofilm formation.

### 3.4. Biocompatibility Performance

**Figure 6:**
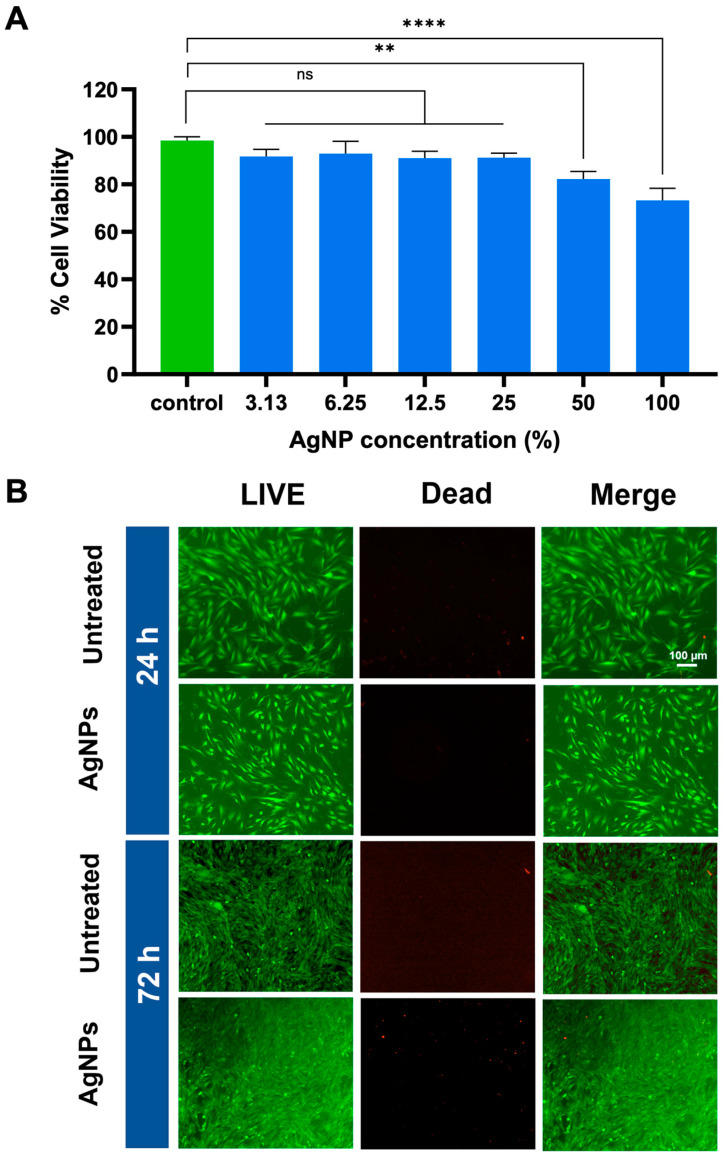
(**A**) Cell viability at different AgNP concentrations compared to the untreated control (green) tested at 24 h. Each value represents mean ± SD (n = 3). ** indicates a difference at *p* < 0.001 and **** at *p* < 0.0001, and “ns” is no significance. (**B**) Fluorescence microscopy images showing the viability of MG63 cells cultured on AgNP/PAA-coated Ti implants and untreated Ti (control) at two time points: 24 h and 72 h. The assay distinguishes live (green) and dead (red) cells to assess biocompatibility and cytotoxicity.

AgNPs have gained significant attention due to their potent antimicrobial properties and applications in biomedical fields. However, their safe use requires a thorough evaluation of their cytotoxicity, particularly when stabilized with biocompatible agents such as polyvinylpyrrolidone (PVP). The cell viability (%) of cells treated with two-fold serial dilutions of PVP-stabilized AgNPs was assessed using an MTT assay, which is a standard method to assess cellular metabolic activity and viability. In this experiment, the 24.5 µg/mL AgNP concentrations were defined as 100 percent in the MTT assay. MG63 cell lines were cultured under standard conditions and treated with different concentrations of AgNPs. The percentage of cell viability after 24 h of exposure to various concentrations of AgNP coatings (3.13%, 6.25%, 12.5%, 25%, 50%, and 100%) compared to the untreated control (green bar) is shown in Figure 6A. The cells treated with AgNPs maintained high viability (>70%) across all tested concentrations (3.13–100%), indicating minimal cytotoxic effects of AgNPs on these cells. Our study confirmed that lower concentrations exhibited reduced cytotoxicity, while also following the observed trend of decreasing viability at higher concentrations [26]. This suggests that the PVP-stabilized formulation effectively reduces the potential cytotoxicity of AgNPs, likely due to the biocompatibility provided by the PVP [47,48]. The MTT assay results show that PVP-stabilized AgNPs exhibited low toxicity to the cells in the short term (24 h), making them suitable for biomedical applications. At low concentrations, AgNPs selectively target MRSA via anionic phospholipids and teichoic acids, destabilizing membranes and causing ROS damage. In contrast, the MG63 cells resist toxicity with robust membranes and antioxidant defenses [49,50].

To further evaluate biocompatibility, we conducted a qualitative analysis of 15-cycle AgNP/PAA-coated Ti plates. Using the LIVE/DEAD staining method, we compared the coated and uncoated Ti plates to assess their biocompatibility. The results presented in Figure 6B highlight the viability of MG63 cells cultured on the implants at two time points: 24 h (short-term response) and 72 h (long-term response). The assay distinguishes live cells (green fluorescence) from dead cells (red fluorescence) to evaluate cytotoxicity and cell viability. In the control group (uncoated Ti implants), predominantly green fluorescence was observed at both 24 and 72 h, indicating high cell viability and minimal cytotoxicity. Similarly, AgNP/PAA-coated Ti implants showed consistent green fluorescence with sparse or negligible red fluorescence at both time points. This suggests that the coating does not induce significant cytotoxicity over time. Additionally, the cells cultured on the coated implants exhibited elongated and evenly distributed morphology, indicative of healthy MG63 cell adhesion and proliferation. The dominance of green fluorescence at both time points confirms the robust biocompatibility and cytocompatibility of the AgNP/PAA-coated surfaces. This indicates that the coating effectively supports cellular attachment and growth. The LIVE/DEAD assay results further demonstrate that the AgNP coating exhibited antimicrobial efficacy while remaining non-toxic to MG63 cells. These findings suggest that the coating possesses significant biocompatibility, underscoring its potential for use in biomedical coating applications.

## 4. Conclusions

In this study, we successfully developed multilayered AgNP/PAA-coated Ti implants using a layer-by-layer dip coating technique. AgNPs were synthesized using PVP as a stabilizing agent and coated layer-by-layer with PAA using our in-house dip coater. Both PAA and PVP served as biocompatible materials, showcasing their potential in controlled and tailored environments. Key parameters, including dipping rate, evaporation time, and number of coating cycles, were found to significantly influence coating thickness, thereby affecting antimicrobial efficacy against MRSA. The layer-by-layer approach enabled sustained protection against infections while maintaining excellent biocompatibility. This modified Ti surface demonstrated functionality by effectively eradicating antiadhesive drug-resistant bacteria and releasing killing agents to prevent MRSA-related infections. Our findings provide compelling evidence of the material’s ability to exert antimicrobial and antibiofilm activity without exerting a toxic effect on surrounding cells. This supports the potential of AgNPs as a promising alternative to conventional antibiotics, offering multiple mechanisms of action, strong efficacy against biofilms, and bactericidal effectiveness at low concentrations. This developed coating strategy offers a promising solution for preventing MRSA-related orthopedic infections. However, challenges such as the lack of inherent antibacterial properties and occasional biodegradability highlight areas for further improvement. Future studies should focus on long-term research to investigate the biocompatibility and efficacy of silver ions released from coatings on Ti and evaluate clinical performance. There are still challenges in large-scale production to facilitate the broader adoption of AgNP coatings in advanced implant technologies. Additionally, long-term in vivo studies are needed to explore the effects of the coatings, address potential toxicity concerns, and ensure their safe and effective use in clinical trials, paving the way for their integration into clinical practice.

## Data Availability

The original contributions presented in this study are included in the article. Further inquiries can be directed to the corresponding author(s).
